# A phase I trial investigating pulsatile erlotinib in combination with gemcitabine and oxaliplatin in advanced biliary tract cancers

**DOI:** 10.1007/s10637-016-0406-z

**Published:** 2016-11-16

**Authors:** Laura W. Goff, Dana B. Cardin, Jennifer G. Whisenant, Liping Du, Tatsuki Koyama, Kimberly B. Dahlman, Safia N. Salaria, Ruth T. Young, Kristen K. Ciombor, Jill Gilbert, Stephen James Smith, Emily Chan, Jordan Berlin

**Affiliations:** 1 Vanderbilt-Ingram Cancer Center, Vanderbilt University Medical Center, 2220 Pierce Avenue, PRB 777, Nashville, TN 37232 USA; 2 Department of Medicine, Vanderbilt University Medical Center, Nashville, TN USA; 3 Department of Biostatistics, Vanderbilt University Medical Center, Nashville, TN USA; 4 Center for Quantitative Sciences, Vanderbilt University Medical Center, Nashville, TN USA; 5 Department of Cancer Biology, Vanderbilt University, Nashville, TN USA; 6 Vanderbilt-Ingram Cancer Center at Cool Springs, Franklin, TN USA; 7 Ohio State University, Columbus, OH USA

**Keywords:** Biliary tract cancers, Pulsatile erlotinib, Phase Ib trial, Gemcitabine and oxaliplatin

## Abstract

Advanced biliary tract cancers (ABTC) are among the deadliest malignancies with limited treatment options after progression on standard-of-care chemotherapy, which includes gemcitabine (GEM) and oxaliplatin (OX). The epidermal growth factor receptor inhibitor erlotinib has been explored in ABTC with modest efficacy. Erlotinib given continuously may antagonize the action of chemotherapy against cycling tumor cells, but pulsatile dosing of erlotinib with chemotherapy may improve efficacy. The purpose of this study was to assess the safety of pulsatile erlotinib with GEMOX. This was a single-institution phase Ib study that enrolled adult patients with unresectable or metastatic biliary tract, pancreas, duodenal, or ampullary carcinomas that have not received any prior treatment for their disease. Dose escalation followed a standard 3 + 3 design, and dose-limiting toxicities (DLTs) were any treatment-related, first course non-hematologic grade ≥ 3 toxicity, except nausea/vomiting, or grade 4 hematologic toxicity. A dose expansion cohort in ABTC was treated at the MTD. Twenty-eight patients were enrolled and 4 dose levels were explored. The MTD was erlotinib 150 mg + GEM 800 mg/m^2^ + OX 85 mg/m^2^. DLTs were diarrhea and anemia. Most frequent toxicities were nausea (78 %), fatigue (71 %), neuropathy (68 %), and diarrhea (61 %), predominantly grade 1–2. In the ABTC patients, the objective response and disease control rates were 29 % and 94 %, respectively, and median overall survival was 18 months. Erlotinib plus GEMOX was well tolerated. Encouraging anti-tumor activity was seen as evidenced by a high disease control rate and longer median OS than standard chemotherapy in the patients with ABTC.

**Electronic supplementary material:**

The online version of this article (doi:10.1007/s10637-016-0406-z) contains supplementary material, which is available to authorized users.

## Introduction

Approximately 7500 new cases of advanced biliary tract cancer (ABTC) will be diagnosed each year in the United States [1]. These are generally divided into intrahepatic cholangiocarcinoma, extrahepatic cholangiocarcinoma, and cancer of the gallbladder. In addition, ampulla of Vater cancer is variably included with biliary cancers. These cancers frequently present in a stage too advanced for surgical resection, and a majority of the patients with operable disease will have recurrence after complete resection. Several chemotherapy agents have been evaluated in biliary tract cancer patients, with a gemcitabine backbone emerging as a standard [2]. A phase II trial of single-agent gemcitabine observed an objective response rate of 22 % and a disease control rate of 50 % [3]. In a phase II study of first-line therapy, the combination of gemcitabine and oxaliplatin (GEMOX) was well tolerated and resulted in an objective response rate of 35 % and stable disease in 26 % [4]. Despite the modest success with gemcitabine combinations, biliary cancers remain among the deadliest malignancies [5, 6]. Therefore, the development of new therapeutic regimens to improve treatment efficacy in this patient population is warranted.

The epidermal growth factor receptor (EGFR) is part of a complex series of cellular signaling pathways that lead to increased cell proliferation, motility, survival, and angiogenesis, all of which can lead to tumor growth and progression [7]. EGFR expression is increased in a majority of bile duct cancers along with one of its ligands, TGF-alpha [8]. Additionally, HER2, which can dimerize with EGFR thus activating downstream signaling pathways, is overexpressed (immunohistochemical staining ≥2) in 15–43 % of biliary cancers [9–11]. Thus, there has been hope for the development of EGFR-directed therapies for the treatment of ABTC [12–14]. Erlotinib, an orally active tyrosine kinase inhibitor (TKI) of EGFR, is currently FDA approved for use in non-small cell lung cancer, as well as in pancreatic cancer when administered in combination with gemcitabine. In a study of 42 patients with either unresectable or metastatic biliary cancer being treated with daily oral doses of erlotinib, 7 % achieved an overall confirmed response, 43 % achieved stable disease, and the median OS was 7.5 months [14].

Preclinical data from mouse xenograft models suggested that pulsed gefitinib (another EGFR TKI) before paclitaxel caused significantly more tumor regression than continuous gefitinib dosing in combination with paclitaxel [15]. When pancreas, gastric, and colon cancer cell lines were treated with combinations of gemcitabine with flavopiridol, an agent that induces G1/S cell cycle arrest, maximal antitumor effect was observed with the combination of gemcitabine followed by flavopiridol, whereas the reverse sequence showed no synergy [16]. As erlotinib also induces G1/S arrest, we hypothesized that erlotinib may exhibit sequence-specific synergy with gemcitabine in a similar manner, such that pulsatile dosing after chemotherapy with adequate washout prior to repeat dosing would achieve improved efficacy. Therefore, our primary objective in this phase Ib study was to determine the maximum tolerated dose (MTD) and recommended phase II dose (RP2D) of pulsatile erlotinib in combination with GEMOX. A secondary objective was to describe any anti-tumor activity associated with treatment in patients with ABTC. Exploratory objectives included an investigation of the relationship between clinical response and the expression levels of the cytoskeleton protein vimentin and the cell-cell adhesion protein E-cadherin. Furthermore, given the evidence that patients with *KRAS* mutations have a poorer response to EGFR-directed therapies [17, 18] and that biliary tract cancers have a spectrum of mutations in *EGFR* and its downstream signaling pathways, including *KRAS* and *PIK3CA* [19–23], we evaluated the potential relationship between mutational status and clinical outcome.

## Materials and methods

### Ethics statement

This trial was conducted in accordance with Good Clinical Practice and the Declaration of Helsinki. The study protocol and informed consent document were approved by our Institutional Review Board. The study was registered through ClinicalTrials.gov (NCT00987766).

### Study design and patient selection

This single-institution, open-label, phase Ib study of GEMOX with erlotinib was conducted in patients with previously untreated advanced adenocarcinoma of the biliary tract, pancreas, duodenum, or ampulla using a standard 3 + 3 design. The primary objective was to determine the MTD and RP2D of pulsatile erlotinib in combination with GEMOX. Secondary objectives were to describe any anti-tumor activity associated with treatment and to correlate response with tumor cell expression of E-cadherin and vimentin, and *KRAS* and *EGFR* mutational status. An expanded cohort (*n* = 10) of patients with ABTC treated at the MTD was included to further describe anti-tumor activity.

Adult patients with unresectable or metastatic cancers that were histologically or cytologically confirmed to be biliary tract, pancreas, duodenal, or ampullary carcinomas were included. Patients must not have had prior chemotherapy or prior EGFR-targeted therapy for their disease. Patients were required to have an Eastern Cooperative Oncology Group performance status (ECOG PS) of 0, 1, or 2 with adequate bone marrow, renal, and hepatic function defined as pretreatment bilirubin less than 2.5 times the upper limit of normal (ULN) and hepatic transaminases less than 2.5 times ULN or less than 5 times ULN if liver metastases were present. Patients with central nervous system metastases or other recent cancers were excluded. Additionally, patients with uncontrolled infection, significant neuropathy, or any other concurrent medical condition that would make the patient an inappropriate candidate for study enrollment were not included.

### Treatment

The dose escalation schema (Table 1) began with gemcitabine (800 mg/m^2^) as a 10 mg/m^2^/min infusion on day 1, followed by oxaliplatin (85 mg/m^2^) as a 2 h infusion on day 2 every two weeks. A fixed dose rate (FDR) of 10 mg/m^2^/min for gemcitabine was chosen based on the prior phase II study of GEMOX in ABTC [4]. The FDR for gemcitabine was used for all dose levels. The starting dose for erlotinib was 50 mg given orally once daily for 5 days on days 3–8. Dose escalation was planned for groups of 3 patients until the MTD was established in a standard 3 + 3 design. No intrapatient dose escalation was allowed.

**Table 1:** Dose escalation schema

Dose Level	Erlotinib	Gemcitabline	Oxaliplatin	Course 1 (two 14-day cycles) toxicity
0	50 mg	800 mg/m^2^	85 mg/m^2^	4 patients (3 evaluable);
				0/3 patients with DLT
1	75 mg	800 mg/m^2^	85 mg/m^2^	5 patients (3 evaluable);
				0/3 patients with DLT
2	100 mg	800 mg/m^2^	85 mg/m^2^	3 patients;
				0/3 patients with DLT
3	150 mg	800 mg/m^2^	85 mg/m^2^	3 patients;
				0/3 patients with DLT^a^
4	150 mg	1000 mg/m^2^	85 mg/m^2^	2 patients;
				2/2 patients with DLT^b^

^a^Additional 10 patients treated at this dose level in expansion cohort

^b^Grade 3 diarrhea, grade 4 anemia

The dose limiting toxicity (DLT) was defined as any treatment-related toxicity occurring in the first two 14-day cycles that was Grade ≥ 3 except for nausea/vomiting or hematologic toxicity. For hematologic toxicity, grade 4 anemia was a DLT. Grade 4 neutropenia lasting for more than five days or grade 4 neutropenia of any duration associated with a fever ≥38.5 °C or infection was considered a DLT. Grade 4 thrombocytopenia was also dose limiting. For patients with grade 3 or 4 nausea or vomiting not amenable to maximal antiemetic therapy, the dose level could be reduced 1 or 2 levels at the discretion of the investigator. Treatment on hold due to toxicity for longer than three weeks was also considered a DLT.

The MTD was said to be exceeded when at least 2 of 6 patients experienced a DLT. MTD is therefore the dose at which 0 or 1 of 6 experience a DLT with the next higher dose level provoking a DLT in 2 of 6 or 2 of 3 patients. Once the DLT was reached, a cohort of 10 additional patients with ABTC was enrolled and treated at the MTD in order to confirm safety.

### Assessments

#### Safety

Toxicity assessments were performed each cycle and graded according to the NCI Common Toxicity Criteria, Version 3.0 (CTCAE v3). All patients who received any chemotherapy on this study were considered evaluable for safety. Patients removed from the study during the first four weeks of treatment for reasons other than progressive disease or drug-related adverse events were considered not evaluable for DLT and were replaced.

#### Anti-tumor activity

All eligible patients who received at least two 14-day cycles of therapy on this study were evaluable for anti-tumor activity. Patients removed from study before four weeks for progressive disease or drug related adverse event were also evaluable for efficacy. Tumor evaluation by radiographic examination was performed every 8 weeks, using Response Evaluation Criteria in Solid Tumors (RECIST 1.0). Reassessment of the extent of tumor was done by the same imaging method used to establish baseline tumor measurements.

#### Laboratory correlates

A lung triplex sizing assay was performed on pre-treatment archived tumor specimens (if available) to assess the presence of *EGFR* and *HER2* exon 20 insertions and *EGFR* exon 19 deletion [24]. Additionally, the sequences of *KRAS* exons 2 and 3 and *EGFR* exons 20 and 21 were evaluated for hotspot mutations using PCR and Sanger sequencing. Lastly, immunohistochemical (IHC) staining for E-cadherin and vimentin was performed. A detailed description of the methods for each correlate analysis can be found in the supplemental methods section.

#### Statistical analysis

Patient characteristics were summarized using descriptive statistics including frequencies and medians. Safety and efficacy data were summarized for all patients enrolled onto the study, and for only those patients with ABTC. The objective response rate (ORR) was calculated as the relative frequency of patients who had complete or partial response among all evaluable patients. Confidence intervals were estimated using the Wilson method, and survival function was estimated using Kaplan-Meier method.

## Results

### Patient characteristics

**Fig. 1:**
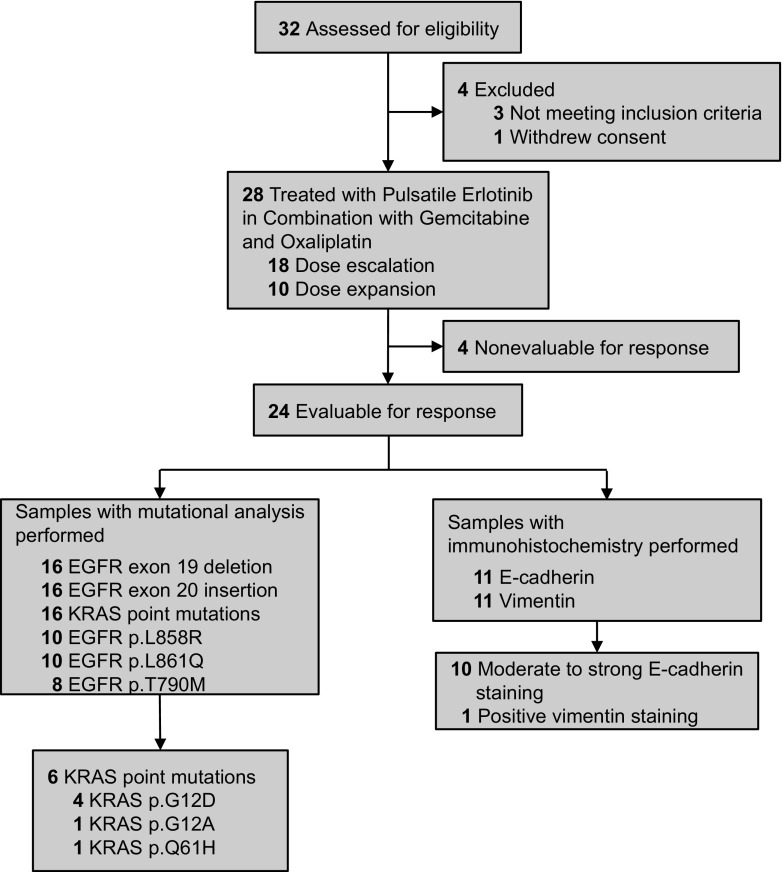
Clinical trial flow diagram that depicts the number of patients that were consented, received study therapy, and evaluable for response. The diagram also depicts the number of samples tested for the correlative analysis, as well as the correlative results

Twenty-eight patients were enrolled between January 2010 and April 2013 (Table 2), 18 patients were enrolled during the dose escalation with an additional 10 patients with ABTC, who were treated at the MTD (Fig. 1). Approximately half of the patients had intrahepatic cholangiocarcinoma (*n* = 13), while five patients had extrahepatic cholangiocarcinoma, eight patients had pancreatic cancer, and one patient each had cancer of the gallbladder and ampulla. All of the patients had a good performance status (ECOG PS of 1 or better) at time of enrollment. The median age of the participants was 61.5 years.

**Table 2:** Patient characteristics

Data	Results
Median age (range)	61.5 (36–85)
Primary tumor type	
Intrahepatic Cholangiocarcinoma	13
Extrahepatic Cholangiocarcinoma	5
Gallbladder	1
Ampulla of Vater	1
Pancreas	8
Disease Status	
Locally Advanced	8
Metastatic	20
Median CA 19–9 Level (range)	482.5 (1–27,973)
PS (ECOG)/no. patients	
0	6
1	22
Gender	
Male	16
Female	12

*PS* Performance status; *ECOG* Eastern Cooperative Oncology Group

### Treatment and toxicities

Dose escalation proceeded according to Table 1. At a dose of 150 mg erlotinib with 1000 mg/m^2^ gemcitabine and 85 mg/m^2^ oxaliplatin, two patients experienced a DLT (grade 3 diarrhea and grade 4 anemia). Therefore, the MTD was determined to be 150 mg erlotinib given orally on days 3–8 in combination with gemcitabine 800 mg/m^2^ and oxaliplatin 85 mg/m^2^ administered on cycle day 1 and 2, respectively.

The most frequent toxicities were nausea (78 %), fatigue (71 %), neuropathy (68 %), diarrhea (61 %), rash (57 %), and thrombocytopenia (54 %); a majority of these were grade 1–2. A summary of the significant (≥ grade 3) toxicities is presented in Table 3, with the most frequent being grade 3 fatigue and gastrointestinal toxicities experienced by 3 of 28 and 4 of 28 patients, respectively. One patient experienced grade 4 cerebral ischemia that was determined by the investigators to be possibly related to gemcitabine and erlotinib. As mentioned above, a dose limiting grade 3 diarrhea and grade 4 anemia were experienced by two patients. One patient died while on study and was determined by the investigator to be related to disease progression.

**Table 3:** Toxicity ≥3, Any Cycle (Number of patients treated at that dose level)

	Erlotinib/Gemcitabine/Oxaliplatin				
	50/800/85 (*n* = 4)	75/800/85 (*n* = 5)	100/800/85 (*n* = 3)	150/800/85^a^ (*n* = 14)	150/1000/85 (*n* = 2)
Hematologic					
Anemia	0	0	0	0	1
Leukopenia	0	1	0	1	0
Neutropenia	1	1	0	1	0
Lymphopenia	0	0	2	0	0
Nonhematologic					
Fatigue	0	0	1	2	0
Rash	0	1	0	1	0
Dehydration	0	1	0	0	0
Diarrhea	0	0	0	1	1
Nausea	0	1	0	1	0
Vomiting	0	1	0	0	0
Elevated ALT	0	1	0	1	0
Elevated AST	0	1	0	0	0
Elevated Alk Phos	0	1	0	0	0
Hyperbilirubinemia	0	1	0	0	0
Cerebral Ischemia	0	0	0	1	0

^a^Determined to be the recommended phase II dose

### Efficacy

**Fig. 2:**
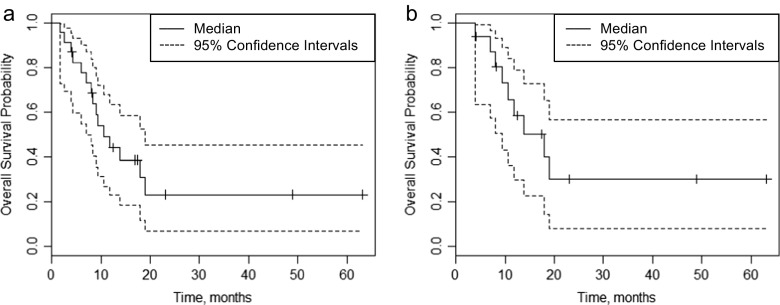
Kaplan-Meier estimates of overall survival stratified by: (A) all patients (*n* = 28) on study and (B) only those patients (*n* = 20) with advanced biliary tract cancers (excluding pancreas). For all patients on study, the median overall survival was 10.6 months (95 % confidence interval, 7.1 to 18.9). The median overall survival in the advanced biliary tract cohort was 18 months (95 % confidence interval, 9.3 to N/A)

Four patients did not complete one course of therapy (i.e., two 14-day cycles) and thus were not evaluable for efficacy endpoints. Two of the four patients withdrew due to patient or physician choice, one withdrew due to an unrelated serious AE, and one patient had disease progression prior to starting therapy. Of the evaluable patients, disease stabilization (SD) occurred in 17 patients, 11 of which had ABTC. A complete response was not observed; however, five patients achieved a partial response (PR) for an ORR of 21 %. Only one patient experienced progressive disease (PD) for an observed disease control rate (DCR) of 92 %. Six patients (25 %) were still on study at six months and were therefore free from progression and unacceptable toxicity. The median OS for all patients was 10.6 months (Fig. 2a; 95 % CI, 7.1 to 18.9). In the ABTC cohort, five of 17 patients achieved a PR for an ORR of 29 %. Eleven patients (65 %) had SD for a DCR of 94 %. The Median OS for the ABTC cohort was 18 months (Fig. 2b; 95 % CI, 9.3 to NE (not estimable given the data)).

### Correlative studies

#### Mutational analysis

None of the tumor tissue samples (*n* = 16) that were evaluated had detectable deletions in *EGFR* exon 19 or insertions in *EGFR* and *HER2* exon 20. Of the 16 tumor tissues sampled for *KRAS* alterations, six tissues (38 %) had nonsynonymous *KRAS* mutations. Two patients with intrahepatic cholangiocarcinoma harbored a *KRAS* mutation in codon 12 (G12A and G12D), and both of these patients achieved a best response of stable disease. Three patients with pancreatic cancer that harbored a *KRAS* mutation in codon 12 (G12D) also achieved a best response of stable disease. Interestingly, an additional pancreatic cancer patient with a *KRAS* mutation had progressive disease, however the mutation was in codon 61 (Q61H). In the patients with wildtype *KRAS*, two ABTC patients achieved a PR, and four ABTC patients and one pancreatic cancer patient achieved SD. No *EGFR* hotspot mutations (L858R, *n* = 10; L861Q, *n* = 10; or T790 M, *n* = 8) were observed in the samples for which tissue was available.

#### Immunohistochemistry

All tumor tissues (*n* = 11) had positive E-cadherin membranous staining with histological score of +1, +2, or +3 with a majority of the samples (*n* = 10) demonstrated moderate (+2) to strong (+3) E-cadherin labeling. Only one patient had positive vimentin staining (histological score = +2), whereas all other evaluated samples stained negative (histological score = 0). No formal statistical associations were evaluated between E-cadherin or vimentin expression and clinical outcomes as there were too few tissue samples (*n* = 8) from patients where disease response was evaluable.

## Discussion

Therapeutic regimens combining chemotherapy with EGFR-targeted agents are an active area of research. Despite the high level of EGFR expression in solid tumors, combining EGFR inhibitors with cytotoxic chemotherapy has not been exceptionally successful in large-scale clinical trials. For example, in the TALENT lung cancer trial, erlotinib was added to gemcitabine and cisplatin without an improvement in OS [25]. Evaluation of 274 patient tumors from the TRIBUTE trial, in which patients with non-small cell lung cancer were treated with carboplatin and paclitaxel with or without erlotinib, showed a trend towards an erlotinib benefit on time to progression but not improved survival [26]. More recently, the BINGO trial in ABTC evaluated GEMOX with and without cetuximab but did not observe an improvement in efficacy [27]. Based on the preclinical evidence suggesting that EGFR tyrosine kinase inhibitors result in a cell cycle arrest rather than inducing apoptosis [28], it is reasonable to hypothesize that antagonist effects with a cytotoxic agent such as chemotherapy may occur. However, based on the preclinical observation that a pulsatile regimen of EGFR-targeted therapy in combination with chemotherapy was more effective [15], we designed the current study to test the hypothesis that erlotinib administered at periodic time points during standard chemotherapy would be a synergistic treatment strategy for patients with ABTC.

The primary objective of this study was to determine the MTD of GEMOX administered in combination with erlotinib. The MTD was determined (and verified in the dose expansion cohort) to be 800 mg/m^2^ GEM, 85 mg/m^2^ OX, and 150 mg erlotinib. When the current study was being developed, we were unaware of the concurrent phase III trial in South Korea that was investigating continuous erlotinib in combination with GEMOX compared to GEMOX alone in ABTC patients [13]. However, despite the slightly different treatment regimens (phase III study: 100 mg erlotinib administered daily, 1000 mg/m^2^ GEM, and 100 mg/m^2^ OX), the toxicity profiles were similar between the two studies suggesting that erlotinib in combination with chemotherapy is well tolerated in patients with ABTC.

It is well known that skin rash is a major side effect of erlotinib, and perhaps pulsatile dosing reduces the incidence of erlotinib-associated skin rash. A meta-analysis of 2911 patients with a variety of solid tumors observed that skin rash associated with single agent erlotinib (150 mg daily) occurred in 75 % of patients [29]. A phase II study that investigated daily erlotinib (150 mg) in combination with bevacizumab (5 mg/kg; Day 1 and 15 of a 28-day cycle) in ABTC reported that 76 % of patients experienced erlotinib-associated skin rash [30]. The total incidence of skin rash in our study was much lower at 57 %, and only 29 % of patients treated at 150 mg developed erlotinib-associated skin rash. Thus, pulsatile dosing (Day 3–8 of a 14-day cycle) of erlotinib could be an alternative treatment strategy to reduce the incidence of rash. Although interval dosing of a TKI, such as erlotinib, is attractive from the context of decreasing the frequency of specific adverse events, the possibility of the “disease flare” phenomenon that occurs after stopping a TKI should not be overlooked. A retrospective study in non-small cell lung cancer investigated the time of development of disease flare after stopping EGFR TKI (either erlotinib or gefitinib) in patients that acquired clinical resistance. This study observed a 23 % flare rate with a median time to disease flare of eight days (range: 3–21 days) [31]. Thus, this phenomenon should be considered when designing future treatment regimens that include interval dosing of erlotinib.

As a secondary objective, we sought to describe any anti-tumor activity associated with the combination of pulsatile erlotinib and GEMOX. As this study completed enrollment, the phase III trial of daily erlotinib plus GEMOX was presented. The South Korean study demonstrated a non-significant improvement in progression-free survival in the cohort treated with erlotinib plus GEMOX compared to GEMOX alone [13]. This lack of significance could be in part a result of erlotinib inducing G1/S cell cycle arrest, instead of apoptosis, which theoretically blocks the subsequent effects of cytotoxic chemotherapy [28]. We observed an ORR of 29 % in the ABTC cohort, which is similar to the 30 % ORR observed by Lee et al. [13]. However, the combination chemotherapy plus pulsatile erlotinib tested in the current study achieved a 94 % DCR compared to 66 % in the phase III study. Furthermore, we observed a provocative OS of 18 months in our ABTC cohort. While these two studies differed in treatment doses, patient demographics, and number of allowed prior therapies, our data suggest that a therapeutic regimen combining chemotherapy with pulsatile dosing of erlotinib may be a better treatment strategy. However, due to the nature of our study design (i.e., phase Ib, 3 + 3 dose-escalation with expansion at the MTD), caution should be taken when interpreting the observed clinical activity due to the small number of patients evaluated in this study. Further prospective trials with larger sample sizes would be needed to confirm activity.

The choice of chemotherapy (e.g., GEMOX) for this study was based on preclinical clinical data showing synergy and clinical efficacy observed in ABTC [4, 32, 33]. Subsequent to the development of our study, gemcitabine plus cisplatin rather than oxaliplatin has emerged as the chemotherapy standard in ABTC. The phase III ABC-02 trial observed an improvement in both ORR (26 % versus 15 %) and DCR (81 % versus 72 %) with gemcitabine plus cisplatin compared to single agent gemcitabine with an excellent toxicity profile [6]. Therefore, one might hypothesize that investigating pulsatile dosing of erlotinib with gemcitabine and cisplatin could be more beneficial. However, the lack of a survival advantage seen by adding erlotinib in the Phase III study with GEMOX limits enthusiasm for exploring this combination with an alternate chemotherapy backbone at the current time. Ideally, as understanding of the complex landscape of the heterogeneous biology of ABTC improves, this could be a future direction, but likely only in a subset of patients.

We had hoped to find a molecularly defined subset of biliary cancers from this study that would be particularly sensitive to EGFR inhibition. Activating mutations in *EGFR* are well-described predictors of response to erlotinib in lung cancer [34–37]. We found no *EGFR* mutations in our patients while the South Korean group found two patients with exon 20 mutations out of 116 total (1.6 %). Although *KRAS* mutational status is an established negative predictor of response to anti-EGFR therapies in colorectal cancer [38, 39], it is currently unknown if this status predicts response in patients with ABTC. The South Korean phase III study suggested a survival benefit with the addition of erlotinib to GEMOX in patients with wild-type *KRAS* [19]. Of the 11 ABTC patients in our study that had available tissue for *KRAS* testing, two with wild-type *KRAS* achieved a partial response, whereas the two ABTC patients with a *KRAS* mutation had stable disease. Thus, our results are in concordance with the previous phase III study suggesting a trend towards a survival advantage when chemotherapy is combined with anti-EGFR therapy in ABTC patients with wild-type *KRAS*. However, contradictory to the observed trends in survival for wild-type *KRAS* patients, three of the six patients with a *KRAS* mutation in the South Korean phase III study still responded to the combination therapy [19]. Furthermore, Gruenberger et al. investigated GEMOX in combination with cetuximab and reported that two of the three patients with *KRAS* mutations achieved a partial response [12]. These contradictory findings between *KRAS* mutation and response suggest that anti-EGFR therapies or, more likely, GEMOX might be beneficial irrespective of *KRAS* mutation in ABTC; however, further investigation is still needed to fully characterize the prognostic indication of a *KRAS* mutation in advanced biliary cancers. The TCGA analysis of biliary cancers demonstrates that there is extensive molecular heterogeneity of these tumors [40]. At the present time it is unclear what role, if any, EGFR inhibitors such as erlotinib may have in emerging subsets such as *FGFR* mutant, *IDH* mutant or microsatellite instability high tumors where more specific targeted or immunotherapy has demonstrated response [41–43].

In conclusion, pulsatile erlotinib with GEMOX administered at the established MTD was well tolerated with an acceptable toxicity profile in patients with advanced pancreatic and biliary tract cancers. Additionally, this treatment combination resulted in encouraging anti-tumor activity as evidenced by a high disease control rate and longer median OS in an ABTC cohort.

## Electronic supplementary material

ESM 1 (DOCX 24 kb)
