# Supplementary material for: A phase I trial investigating pulsatile erlotinib in combination with gemcitabine and oxaliplatin in advanced biliary tract cancers
Source: Invest New Drugs. 2016 Nov 16;35(1):95–104. doi: 10.1007/s10637-016-0406-z (PMC5306261; doi:10.1007/s10637-016-0406-z)
Supplement: Supplementary file 1 — (DOCX 24 kb) [file 10637_2016_406_MOESM1_ESM.docx]

**A Phase I Trial Investigating Pulsatile Erlotinib in Combination with Gemcitabine and Oxaliplatin in Advanced Biliary Tract Cancers**

Laura W. Goff, MD, Dana B. Cardin, MD, Jennifer G. Whisenant, PhD, Liping Du,Tatsuki Koyama, PhD, Kimberly B. Dahlman, PhD, Safia N. Salaria, MD, Ruth T. Young, MD, Kristen K. Ciombor, MD, Jill Gilbert, MD, Stephen James Smith, MD, Emily Chan, MD, PhD, Jordan Berlin, MD

**Corresponding author:**

Laura W. Goff, MD

Vanderbilt-Ingram Cancer Center

[Laura.goff@vanderbilt.edu](mailto:Laura.goff@vanderbilt.edu)

**SUPPLEMENTARY METHODS**

**Lung Triplex Assay**

A lung triplex sizing assay was used to assess insertions and deletions in *EGFR* exon 19 and 20 and *HER2* exon 20. For this assay, DNA was extracted using QIAamp DNA FFPE Tissue Kit (Qiagen, cat. # 56404). DNA concentration was measured using Quant-iT Picogreen dsDNA Reagent (Invitrogen, cat. # P7591). Lung Triplex Sizing Assay reactions were prepared as previously described,^23^ using HotStar Taq Master Mix (Qiagen, cat. # 203443) with 3 ng of DNA (3 ul at 1 ng/ul). PCR cycling conditions were as follows: 15' @ 95C, followed by 40 cycles of (30" @ 94C, 30" @ 60C, 1' @ 72C), followed by 10' @ 72C. DNA oligonucleotides were obtained from Integrated DNA Technologies. The following PCR primers were used: EGFR_Ex19_FWD1 gcaccatctcacaattgccagtta, EGFR_Ex19_REV1 /6FAM/ aaaaggtgggcctgaggttca; EGFR_Ex20ins_FWD tcttcacctggaaggggtcc, EGFR_Ex20ins_REV /HEX/ acggtggaggtgaggcagat; ERBB2_Ex20_FWD accgtgcccggcctaatctt, ERBB2_Ex20_REV /HEX/ tcaggcagatgcccagaagg. PCR products were diluted 1:100 in water. Genescan 400 HD ROX Size Standard (Applied Biosystems, cat. # 402985) was diluted 1:60 in H-Di formamide (Applied Biosystems, cat. # 4311320). 2 ul of diluted PCR product was mixed with 10 ul Hi-Di/Size Standard mix in preparation for fragment analysis. Results were visualized using Genemapper software v4.0 (Applied Biosystems).

**PCR and Sanger sequencing**

Hotspot mutations in *KRAS* exons 2 and 3 and *EGFR* exons 20 and 21 were evaluated using PCR and Sanger sequencing. For these assays, DNA was extracted using QIAamp DNA FFPE Tissue Kit (Qiagen, cat. # 56404). DNA concentration was measured using Quant-iT Picogreen dsDNA Reagent (Invitrogen, cat. # P7591). PCR was performed using HotStar Taq Master Mix (Qiagen, cat. # 203443) with 20 ng of DNA (4 ul at 5 ng/ul). For EGFR exon 20, *EGFR* exon 21, and *KRAS* exon 2, PCR cycling conditions were as follows: 15' @ 95C, followed by 40 cycles of (30" @ 95C, 30" @ 58C, 1' @ 72C), followed by 5' @ 72C. For *KRAS* exon 3, PCR cycling conditions were as follows: 15' @ 95C, followed by 40 cycles of (30" @ 95C, 30" @ 55C, 1' @ 72C), followed by 5' @ 72C. PCR products were run on a 1% agarose gel. Discrete bands of the appropriate sizes were cut out and PCR products were purified using a QIAquick Gel Extraction Kit (Qiagen, cat. # 28704) in preparation for Sanger sequencing. DNA oligonucleotides were obtained from Integrated DNA Technologies. Primers were designed using Primer3 software v0.4.0 (Whitehead Institute for Biomedical Research, Cambridge, MA) (1, 2). The following PCR primers were used: EGFR_ex20_225_M13F(-41) **ggttttcccagtcacgac**cacactgacgtgcctctcc, EGFR_ex20_225_M13R **caggaaacagctatgac**ccctgattacctttgcgatct; EGFR_ex21_187_M13F41 **ggttttcccagtcacgac**ggcatgaactacttggaggac, EGFR_ex21_187_M13R **caggaaacagctatgac**aatgctggctgacctaaagc; KRAS_ex2_snapseq_M13F41 **ggttttcccagtcacgac**tcattatttttattataaggcctgctg, KRAS_ex2_snapseq_M13R **caggaaacagctatgac**agaatggtcctgcaccagtaa; DCKRAS61_f ccagactgtgtttctcccttc, DCKRAS61_M13R **caggaaacagctatgac**aaagaaagccctccccagt. M13 tags (M13F/-41 and M13R) are in bold. Sequencing traces were visualized using Bioedit software v7.2.5 (Ibis Biosciences).

**Immunohistochemistry**

For immunohistochemical (IHC) staining of E-cadherin and vimentin, unstained slides were cut, deparaffinized, and submitted to heat-induced antigen retrieval on a Leica Bond Max IHC Stainer using Leica’s Bond Epitope Retrieval Solution 2 Novacostra (Leica Biosystems, cat# AR9640) for 15 min for E-Cadherin staining and using Leica’s Epitope Retrieval 1 solution (Leica Biosystems, cat# AR9961) for 20 min for vimentin staining. Anti-E-Cadherin (R&D Systems, cat# AF748) was used at a 1:500 dilution for 1 h, followed by incubation with biotinylated anti-goat (Vector Laboratories, Inc., cat# BA-5000) secondary antibody (1:200) for 30 min. For vimentin staining, slides were incubated with ready-to-use anti-vimentin (Leica Biosystems, cat# PA0033) for 15 minutes. All antibody incubations were done at room temperature. The Bond Polymer Refine Detection Novacostra System (Leica Biosystems, cat# DS9800) was used for visualization of both sets of slides. Slides were then dehydrated, cleared, and coverslipped.
